# Validation of the Argentine version of the Memory Binding Test (MBT) for Early Detection of Mild Cognitive Impairment

**DOI:** 10.1590/S1980-5764-2016DN1003008

**Published:** 2016

**Authors:** Fabian Roman, Mónica Iturry, Galeno Rojas, Ernesto Barceló, Herman Buschke, Ricardo F. Allegri

**Affiliations:** 1 Center for Aging and Memory Research, Hospital General Abel Zubizarreta (GCABA), Buenos Aires, Argentina.; 2 University Corporation of the Coast (CUC), Barranquilla, Colombia.; 3 Albert Einstein College of Medicine, Yeshiva University, New York, USA.; 4 Memory and Aging Center, Institute for Neurological Research "Raúl Carrea" (FLENI), Buenos Aires, Argentina.

**Keywords:** episodic memory, Alzheimer's disease, early detection, forgetfulness, screening, assessment

## Abstract

**Background:**

"Forgetfulness" is frequent in normal aging and characteristic of the early stages of dementia syndromes. The episodic memory test is central for detecting amnestic mild cognitive impairment (MCI). The Memory Binding Test (MBT) is a simple, easy and brief memory test to detect the early stage of episodic memory impairment.

**Objective:**

To validate the Argentine version of the MBT in a Latin American population and to estimate the diagnostic accuracy as a tool for early detection of MCI.

**Methods:**

88 subjects (46 healthy controls and 42 patients with amnestic MCI) matched for age and educational level were evaluated by an extensive neuropsychological battery and the memory binding test.

**Results:**

A significantly better performance was detected in the control group; all MBT scales were predictive of MCI diagnosis (p<.01). The MBT showed high sensitivity (69%) and high specificity (88%), with a PPV of 93% and a NPV of 55% for associative paired recall. A statistically significant difference (c^2^=14,164, p<.001) was obtained when comparing the area under the curve (AUC) of the MBT (0.88) and the MMSE (0.70).

**Conclusion:**

The Argentine version of the MBT correlated significantly with the MMSE and the memory battery and is a useful tool in the detection of MCI. The operating characteristics of the MBT are well suited, surpassing other tests commonly used for detecting MCI.

## RESUMO

"Esquecimento" é queixa frequente no envelhecimento normal e também ocorre nos primeiros estágios de síndromes demenciais. Testes de memória episódica são fundamentais para detectar comprometimento cognitivo amnéstico (CCL). O teste de Memória Associativa *(Memory Binding Test-MBT)* é um teste fácil e breve para detectar a fase inicial de perda de memória episódica.

**Objetivo:**

Validar a versão argentina do MBT e estimar a sua acurácia como instrumento diagnóstico para a detecção precoce do CCL.

**Métodos:**

88 indivíduos (46 controles saudáveis ​​e 42 pacientes com CCL amnéstico), emparelhados por idade e nível educacional, foram avaliados com extensa bateria neuropsicológica e o MBT.

**Resultados:**

Um desempenho significativamente melhor foi detectada no grupo controle; todas as escalas do MBT foram preditivas do diagnóstico de CCL (p<0,01). O MBT apresentou alta sensibilidade (69%) e alta especificidade (88%), com valor preditivo (VP) positivo de 93% e e VP negativo de 55% para a recordação dos itens associados *(associative paired recall).* Diferença estatisticamente significativa (c^2^=14,164, p<0,001) foi obtida quando foram comparadas as áreas sob as curvas (AUC) do MBT (0,88) e o Mini-Exame do Estado Mental (MEEM) (0,70).

**Conclusão:**

A versão argentina do MBT correlacionou-se significativamente com o MEEM e com a bateria de memória e é uma ferramenta útil na detecção de CCL. As características operacionais do MBT são bem adequadas, superando outros testes usualmente utilizados para a detecção de CCL.

## INTRODUCTION

"Forgetfulness" is very frequent in normal aging, but is also characteristic of the early stages of dementia syndromes. This situation has prompted neuroscientists for more than 40 years to try to differentiate between age-related memory decline and memory impairment associated with neurodegenerative processes. The low detection rate of Mild Cognitive Impairment (MCI), the use of low-sensitivity screening tests, and the high prevalence of Alzheimer's disease (AD), emphasize the need to develop simple, sensitive and specific instruments for early detection of MCI. There is now consensus that improving the early detection of Alzheimer's disease, before dementia develops, is an urgent priority.^1^ ^-^ ^3^ This has led to several attempts at early detection, including AD biomarkers (neuroimaging and cerebrospinal fluid biomarkers) with the hope that early treatment may be more effective. However, AD biomarkers are expensive, can be invasive, cannot be repeated easily and cannot be done in all patients. Dubois, Picard and Sarazin (2009) have pointed out that we need a simple screening test to select individuals for AD biomarkers.^4^

In the assessment of patients with dementia, the Mini-Mental State Examination (MMSE) is widely used as a screening instrument but lacks sensitivity for detecting MCI or early stages of dementia.^5^

We need an instrument for early detection of impaired memory that is sensitive, specific, brief, inexpensive, easy to administer and interpret, repeatable and effective for detection of early, pre-symptomatic memory impairment. Early detection of impairment when declining memory is still within the normal range will be useful to evaluate clinical trials of early treatment interventions for primary and secondary prevention, and to select individuals requiring neuroimaging and biomarker studies.

Memory Binding Test. The Memory Binding Test (MBT) is a screening test recently designed by Herman Buschke to detect impairment of memory early in the course of Alzheimer's disease, other dementias, or other causes of memory impairment.^6^ Essential characteristics of the MBT include controlled learning, cued recall, encoding specificity to maximize recall and associative binding, and semantic interference.

*Controlled Learning:* tests of learning and memory generally use uncontrolled learning, in which participants are prompted to remember a list of items, which they can do however they want. Participants may use different strategies at different times, or different strategies from those used by other participants; these strategies are generally unknown to the evaluator. Uncontrolled learning limits the comparison of recall or memory performance across different testing sessions. Controlled learning is needed to ensure that poor recall is indeed due to impairment of memory rather than lack of attention or ineffective strategies. Controlled learning is also necessary for encoding specificity to maximize retrieval. A simple way of controlling learning is to present the items to be remembered and to then ask the participant to identify each item when given the proper cue category. The category cues refer to when the items are already clearly associated: for example, everyone knows that an "Oak" (item) is a 'tree' (category). The advantages of controlled learning are to ensure appropriate care and equal treatment of all items, to induce deep semantic processing of all items, assess memory and learning through the items as memory units, induce all participants to do the same processing, show that the required processing was done, ensure that memory decline is due to memory impairment (not due to lack of attention or ineffective strategies), induce item binding to specific cue, and to provide the basis for encoding specificity to maximize the number of items recalled and the retrieval speed.

*Cued Recall:* Tulving (1974) has indicated that essentially all recall is cued recall, suggesting that cued recall should be appropriate for assessing learning and memory.^7^ ^,^ ^8^ Tulving (1968) pointed out that the memory unit in free recall is not known.^9^ In free recall, the items are not learned independently, but are learned as part of a group containing several items that are remembered together.^10^ ^,^ ^11^ In cued recall, each item is learned and recalled independently, so the number of items retrieved by cued recall can provide an accurate estimate of learning and memory. Furthermore, when cued recall is coordinated with controlled learning by using the same cues for learning and memory, the resulting encoding specificity maximizes recall and retrieval speed.

Maximum retrieval is not achieved by free recall.^12^ Free recall is approximately half of cued recall. Free recall provides a measure of accessibility (what can be retrieved), and cued recall provides a measure of availability (what has been encoded and retained).^13^ Maximum retrieval is necessary to detect memory impairment because "impairment" means maximum retrieval has decreased. For cued recall in the MBT, the cues are presented in the same order used during learning and the participant retrieves the item(s) associated with each cue.^14^ ^-^ ^18^

The procedures for cued recall and controlled learning are essentially the same. Controlled learning and cued recall differ only in the source of the items. In controlled learning, the source of the items is a presentation, while in cued recall, the items originate from memory, using the same cues in the controlled learning and the cued recall. The advantages of cued recall with the same cues used in learning are to ensure attention to and testing of all items, to assess memory and learning by items as legitimate memory units, to control order of recall (all participants recall all items in the same order on all tests, equalizing the interval between learning and recall of all items, obviating the need for interference before recall, and preventing output order interference), to achieve encoding specificity and to maximize the number of items recalled and retrieval speed.

*Encoding specificity:* the principle of "Encoding Specificity"^19^ ^,^ ^20^ is one of the most important concepts that we know about learning and memory.^21^ Encoding specificity is the principle that we can only recall what we have learned, using appropriate recall cues that provide access to available information that was stored during learning. If we can recall only what has been retained using such appropriate cues, learning and memory must be coordinated to provide appropriate recall cues. Encoding specificity means that encoding and retrieval must be coordinated to achieve maximum learning and memory. In the MBT, learning and memory are coordinated using the same cues for learning and for recall.^14^ ^,^ ^15^

*Binding:* assessment of binding^22^ ^,^ ^23^ adds an estimate of associative learning^24^ to the MBT. Binding in the MBT is estimated by learning and recall of two items from the same category in association with a single shared category cue. Binding is shown in the paired recall condition of the MBT by recall of both paired-items from each shared category in the 1^st^ and 2^nd^ lists (pairs); e.g., learning and recall of two items such as "Basketball and Rugby" are associated with the single category of "Sports" shared by both. Binding may be impaired in all older adults with dementia^25^ and may be affected in normal aging, which can serve as an early marker of memory impairment in the prodromal stage of Alzheimer's disease.

*Semantic interference:* important studies by Loewenstein et al, 2007,^26^ using a version of the Fuld Object-memory test^27^ (recall of objects withdrawn from a closed bag), developed the "Semantic Interference Test (SIT)" which shows that "vulnerability to semantic interference" may identify "individuals with MCI likely to progress to dementia".^28^ ^-^ ^32^ The MBT begins with learning and recall of two lists, so that proactive semantic interference can be assessed comparing recall of the second list with recall of the first list. Significantly lower recall of the second list shows proactive semantic interference that may indicate early pre-symptomatic memory impairment.

The aim of the present study was to determine the reliability and validity of the Spanish version of the MBT in South America as a tool for early detection of MCI in a large sample at the Center for Aging and Memory Research of the Hospital Abel Zubizarreta of Buenos Aires City, Argentina.

## METHODS

Design. This was a phase II cross-sectional study of elderly clinical patients attending the Center for Aging and Memory Research of the Hospital General Dr. Abel Zubizarreta, Government of Buenos Aires City, Argentina, between 2013 and 2014. This study was approved by the Local Ethics Committee. After complete explanation of the study to the participants, written informed consent was obtained. 

Study population. The general sample consisted of 88 participants living in the city of Buenos Aires, Argentina, comprising 2 groups, 46 healthy elderly (normal control, NC) group and 42 individuals with Mild cognitive impairment (MCI) group. All participants were monolingual Rioplatense-Spanish speakers.

Participants were recruited from Center for Aging and Memory Research of the Hospital General Dr Abel Zubizarreta selected by convenience sampling of consecutive patients suspected of cognitive impairment. Case controls were caregivers and relatives of patients attending the center without relevant history of neurological, psychiatric or substance abuse problems. 

Procedure. For the evaluation of the population, a semi-structured protocol was used (demographics, personal and family history, current medication, diagnosis, clinical and neurological examination), followed by an extensive neuropsychological battery including the Spanish version of the Mini-Mental State Examination (MMSE)^4^ for Buenos Aires,^33^ the Clock drawing test,^34^ Signoret verbal memory battery,^35^ Trail Making Test,^36^ Verbal Fluency,^37^ Spanish version of the Boston Naming Test,^38^ and the Digit Span forward and backward.^39^ For the study of depressive features, the self-administered scale of depression by Beck et al., 1961 was applied.^40^ Patients were staged by a neurologist according to the Clinical Dementia Rating (CDR).^41^

Based on the above, for the final diagnosis of patients the following MCI criteria^42^ were used: a) memory complaint corroborated by an informant; b) memory impairment (score on the Signoret memory battery below 1.5 SD - age and education-adjusted); c) normal global cognitive function; d) normal activities of daily living; and e) absence of dementia. 

*Memory Binding Test (MBT).* The Memory Binding Test (MBT) is a simple test in which the participant must learn and remember two lists of words by controlled learning (CL) and cued recall (CR). The MBT consists of two lists each containing 16 words belonging to one of 16 different semantic categories. The categories are the same in both lists, but the words of each category in the two lists differ. For example, for "Tree" (category cue) the item in list 1 may be "Oak" whereas the item in list 2 might be "Eucalyptus". These categories are used in the same order for the controlled learning and cued recall in both lists. Five seconds are allowed for each response in controlled learning and cued recall. After cued recall of list 1, list 2 is learned immediately, without delay or interference, as more than six items between learning and recall prevents retrieval from immediate memory.^43^ After recall of list 2, the same category cues are presented again, and the participant is asked to recall both paired-items from both lists for each category cue (in any order). This paired-recall condition provides an estimate of binding of two items to the same category cue, evidenced by the number of pairs recalled from the two lists. The participant is given 10 seconds to recall the two items of both lists associated with each category. After paired recall, the participant is asked to perform "free recall" of the 32 items of both lists (in any order). Free recall can be continued until there is no response for 15 seconds.

*Scoring of MBT.* Since each item (word) is processed independently in controlled learning and cued recall, each item is a memory unit in learning and recall, so that the number of items recalled in the MBT should provide more accurate estimates of learning and retrieval, unlike free recall where the items are learned in chunks^10^ ^,^ ^11^ and the memory unit is unknown.^9^

Basic results on the MBT are: [1] Cue Recall List 1 (CRL1): the number of items recalled from list 1 (range 0-16); [2] Cue Recall List 2 (CRL2): the number of items recalled from list 2 (range 0-16); [3] Cue Recall Both List 1 (CRBL1): the number of items in list 1 recalled from Cue Recall Both Lists (range 0-16); [4] Cue Recall Both List 2 (CRBL 2): the number of items in list 2 recalled from Cue Recall Both Lists (range 0-16); [5] Total Paired Recall (TPR): the number of pairs remembered with category cue (range 0-16); [6] Percentage Paired Recall (PPR): paired-items as a percentage of pairs recalled in paired recall; [7] Free Recall (FR): the number of items recalled by Free Recall at 30, 60, 90, and 120 seconds (range 0-32 ).

Intrusions and repetitions are recorded, but not included in the general results, since intrusions represent guesses and may provide the basis for correction of guessing. 

The total paired recall condition (TPR) is a critical measure that estimates associative binding. A score that incorporates item recall and paired recall can be obtained by adding one point for each item recalled in the paired recall condition plus one point for each pair recalled.

Tounsi et al (1999)^17^ have provided an important measure of "sensitivity to semantic cuing" replicated by Sarazin et al. (2007),^18^ which is essentially an estimate of the proportion of cues that are successful. 

*Spanish translation and transcultural adaptation of the MBT.* Although there is a Spanish version of the MBT for Spain^44^ ^-^ ^46^ and another for Colombia,^47^ some words are infrequent in Argentina (zumo, laton, taburete, alicates, ensaimada, aspirador, colgantes, vitrina, cipres, among others). These could have affected the Encoding Specificity and Binding, two important aspects of the MBT. Therefore, it was decided to produce an Argentine version of the MBT with words that avoid difficulty in the application of the test in the group under study.

Spanish translation and adaptation of the MBT was performed in two stages. The first stage corresponded to the initial translation and adaptation to the Spanish language, this was done by a first team formed by a neuropsychologist, neurologist, and a psychiatrist (all three were bilingual (Spanish natives)). The translation from English into Spanish of all lists of words and their corresponding categories was performed, then the translation from Spanish into English was performed, and if the meaning of the words in both languages ​​was the same the word was included in the list of the Spanish version.

At a second stage, the translated and adapted version was presented to a group of 10 professionals with expertise in clinical and memory problems research. The group comprised 4 neurologists, 2 psychiatrists, and 4 neuropsychologists. This enabled a consensus to be reached on the categories and the items of each list of words used. The group was consulted on the words (translated items) along with their relevance and frequency of use in the study population. At this stage, the categories were adapted to similar terms used in the region, for example in the original test the category "state" was included, whereas in the Argentinian version "province" was used. The items in the categories were also adapted, such as "male name", "Sports", and common names used in the country.

Data analysis. Demographic variables (age and education), neuropsychological battery scores, MMSE, CDT and MBT scores were compared by one-way analyses of variance (ANOVA). The Chi-square test was employed for categorical data (gender). Neuropsychological tests were scored using the raw scores. To assess the frequency and extent of clinically relevant neuropsychological deficits, each test score of the patient group was compared with the respective norm group (control group). Measurements in patients who scored at least 1.5 SD below average compared to age and education-matched controls were considered abnormal. ANOVA were performed to assess the effect of age, sex, and their interaction on different fluency measures, performance over time, and the use of clustering and switching strategies. Partial Eta squared was used to determine the effect size of multivariate and univariate Fs. Compliance with homogeneity of variance assumption was confirmed before analysis. The degree of association between variables was calculated using Pearson's correlation coefficient. Finally, stepwise regression analyses were used to determine the relative contributions of cognitive strategies to explaining the variance in VF measures. The diagnostic accuracy of the scores was assessed by establishing Sensitivity (Sn) and Specificity (Sp) for the optimal cut-offs. Receiver operating characteristic (ROC) curve analysis was performed to evaluate discriminative power between different screening tests. Area under the curve (AUC) was used as a measure of overall ROC curve performance (95% CI). Data processing and statistical analysis were performed using the Statistical Package for the Social Sciences (SPSS) version 15.0, and a statistical significance level below .05 was set.

## RESULTS

Demographic data. 42 patients with MCI and 46 normal control subjects matched for age, education and sex were assessed by an extensive neuropsychological battery (Table 1). A significantly better performance on cognitive assessment in the control group was expected. To confirm this, the distributions of the dependent variables were examined, verifying that most of them did not follow a normal distribution, therefore the Mann-Whitney non-parametric U-test was calculated. All tests showed statistically significant differences, thereby confirming that the controls performed better. 

**Table 1:**
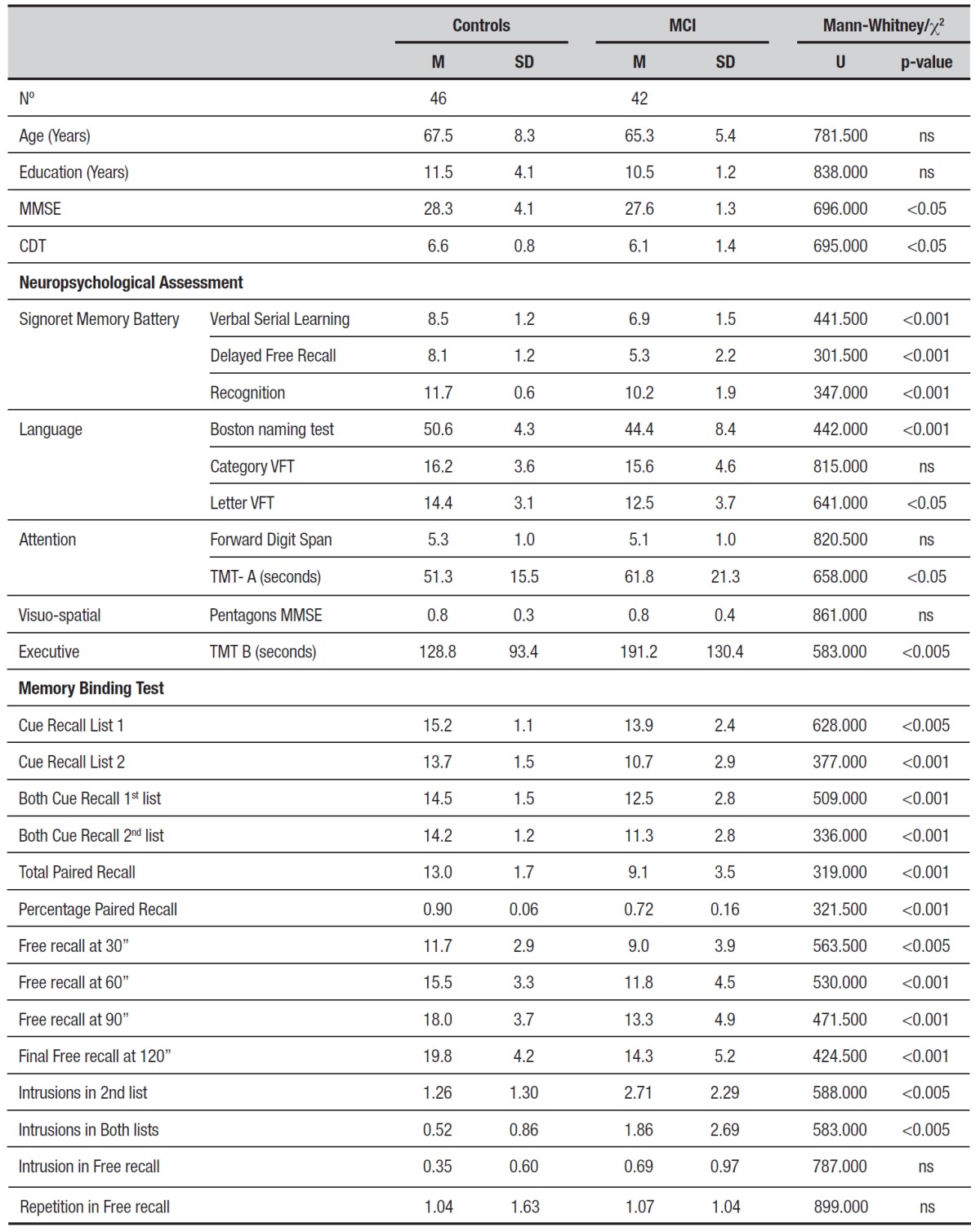
Demographic data and neuropsychological battery.

Values shown represent mean (M) and standard deviation (SD) results except for sample size and sex. Neuropsychological tests scores are represented as raw scores. MCI, mild cognitive impairment; MMSE, Mini-Mental State Examination Test; CDT, Clock Drawing Test; VFT, verbal fluency test; Forward Digit Span Subtest of WMS III; TMT, trail making test A and B. Comparisons simultaneously made among all groups using ANOVA test for all participant features except sex (c^2^ test).

Validation of the MBT. The convergent and discriminative validity between the scales of the MBT and the Signoret memory battery and the MMSE was assessed by Pearson correlations, showing a statistically significant value for all subscales of the MBT (p<0.001).

Operational characteristic of MBT. The operational characteristics of the MBT were studied. To assess sensitivity and specificity, the MCI diagnosis was based on the cognitive performance of the tests (z-score of -1.5 obtained). To assess the z-score, the means and standard deviations were obtained in the control group, based on age and years of education. The positive predictive value and negative predictive value were obtained in each case (Table 2).

**Table 2:** MBT: Operational characteristic and discriminative power.

	Discriminative Power				Operational Characteristic			
	p-value	OR	AUC		Sn	Sp	PPV	NPV
Total Paired Recall	<.001	.527	.889		0.69	0.88	0.93	0.55
Percentage Paired Recall	<.001	.000	.888		0.69	0.90	0.94	0.56
Weighted Recall	<.001	.745	.886		0.66	0.90	0.94	0.53
Final Free Recall 120"	<.001	.712	.857		0.49	0.92	0.93	0.44
Total Intrusions 2^nd^ List	<.001	1.92	.781		0.55	0.90	0.93	0.47
Total Intrusions both Lists	<.001	2.34	.748		0.40	0.88	0.89	0.39

nº=175 (Controls 53; MCI 122); Discriminative power by logistic regressions; AUC: area under the ROC curve; Sn: sensitivity; Sp: specificity; PPV: positive predictive value; NPP: negative predictive value.

Discriminative power of MBT. To evaluate the discriminative power of the test, we calculated logistic regressions to verify the predictive ability of the scales for the diagnosis of MCI (see Table 2). All MBT scales were predictive of MCI diagnosis (p<0.01). They had adequate sensitivity and specificity above the cognitive tests used in this study. The ROC curve of all scales of MBT was calculated. 

Operational characteristic of MBT vs MMSE. Comparison of the operational characteristics of the MBT vs the MMSE and ROC curves was carried out. Sensitivity, specificity, positive predictive value and negative predictive value of the MBT and MMSE were also compared. The area under the curve (AUC) for the MBT was 0.88 (95% CI: 0.84-0.93) and for the MMSE was 0.70 (95% CI 0.62 to 0.78). The test of Hanley and McNeil was used to compare the areas under the curve for both tests revealing a statistically significant difference (c^2^=14,164, p<.001). Therefore, the diagnostic utility of the MBT was significantly higher than that of the MMSE.

Performance on MBT by age and education in the normal healthy population. MBT standardization was performed by age and education in the normal healthy population (a z-score cut-off of -1.5 was used for all tests (Table 3)). 

**Table 3:**
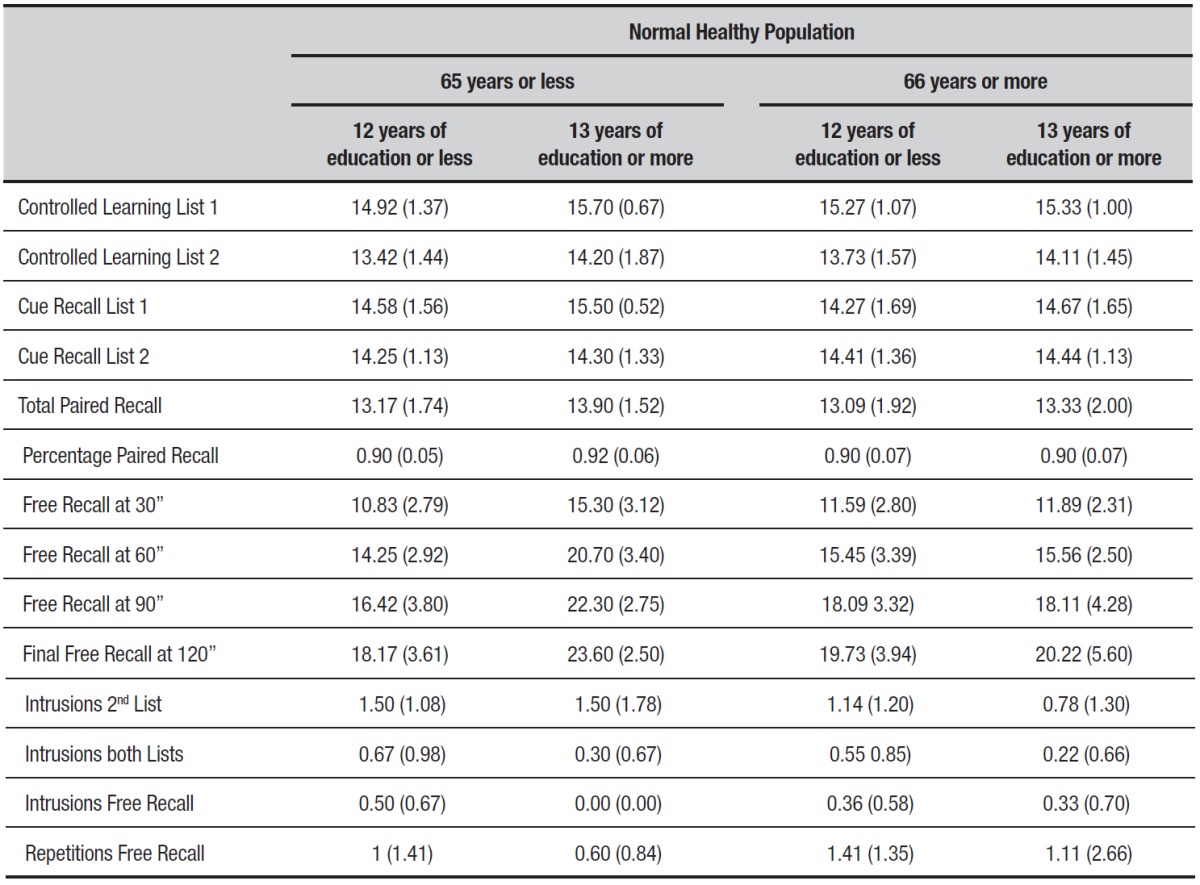
MBT: Norms in normal healthy subjects.

nº=Normal Healthy Population 53. Values shown represent mean (M) and standard deviation (SD).

**Figure 1:**
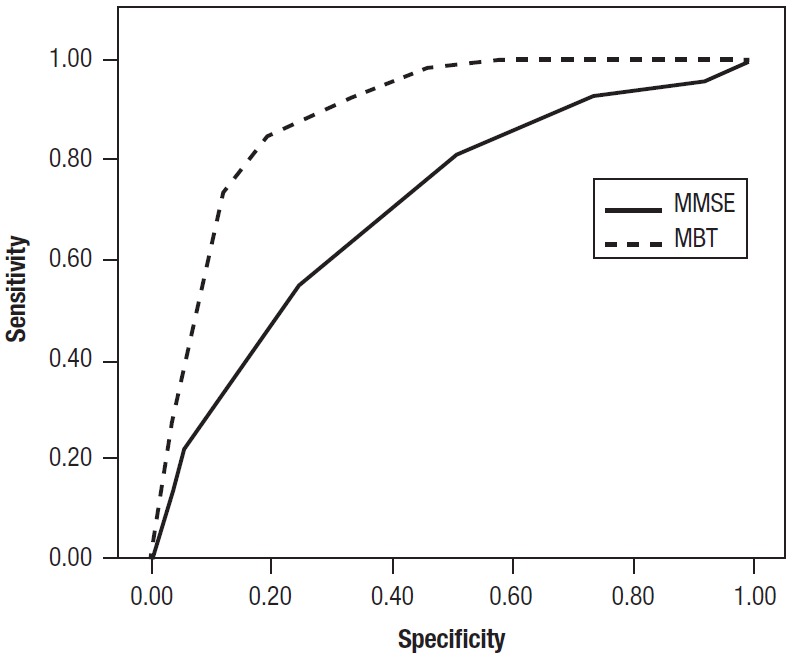
Comparison ROC Curve - MBT (N° Paired recall) vs MMSE.

## DISCUSSION

The aim of this investigation was to evaluate the performance of the Spanish version of the MBT as a screening instrument in a population of MCI and control subjects in Buenos Aires, Argentina. Screening is a "first step" in detecting Alzheimer's disease, and therefore, an important public health and clinical initiative.^48^ Essential features of an effective memory test for screening pre-dementia memory impairment are controlled learning, cued recall, encoding specificity and associative binding. All memory tests are not equal.^49^ Many memory tests are used to detect impairment, but note, when memory impairment is severe enough, almost any memory test can detect impairment. Most memory tests do not use controlled learning; other tests do not induce encoding specificity to maximize retrieval. Many memory tests are based on free recall, which does not maximize retrieval, and others are limited to learning a list of items and do not include binding. Most memory tests are based on comparisons of performance against control subjects matched for age. Consequently, impairment can be detected only when it is severe and therefore late, precluding the early detection of memory impairment in the pre-symptomatic stage. 

The MBT is designed to improve the detection of truly early memory impairment, when declining memory is still within the normal range, by overcoming certain limitations of the Buschke Free and Cue Selective Reminding test (FCSRT).^15^

The MBT consists of two lists of words. By using controlled learning and cued recall, the encoding specificity is created by the associative union of the words to cued category; detecting memory problems through decrease in maximum retrieval. Cued recall provides memory units that can be used to estimate learning and memory and maximizes recall which is essential for detection of memory impairment. Impaired associative binding, evidenced by the low number of pairs remembered through unique cue categories, may provide a marker of early memory impairment in older adults with Alzheimer's disease or other dementias,^50^ and in some older adults without apparent memory impairment tests according to standard tests. The MBT associated binding provides a marker of early memory impairment. The MBT is brief, about 6 minutes for controlled learning and cued recall of list 1 and list 2, and recall of paired-items. The MBT is easily administered, and can be repeated. It is inexpensive and very well accepted by the elderly, as they feel more comfortable with cued recall than free recall. The MBT can serve as a basis to select individuals for AD biomarkers.^51^ ^,^ ^52^

In the present study, the control group and the MCI group were compared using the scales of the MBT and the Signoret memory battery as well as the MMSE, revealing significantly better performance in the control group in statistical terms.

It was observed that all the scales of the MBT were predictive for amnesic MCI diagnosis with adequate sensitivity and specificity above the cognitive tests used in this study.

The MBT had better sensitivity/specificity than the MMSE. The MMSE was unable to detect MCI. In addition, the MBT is a highly sensitive and specific screening test for detecting amnesic MCI. 

A statistically significant difference was apparent when comparing the AUC of the MBT to that of the MMSE, showing the diagnostic utility of the MBT.

A limitation of the present work is that MCI cases were clinically defined but no biomarkers were used to define whether these patients had Alzheimer's disease. The MBT can serve as a basis to select individuals for biomarkers.^51^ ^,^ ^52^

The new criteria for Alzheimer's disease proposed by Dubois et al.^2^ ^,^ ^4^ suggested that the diagnosis of Alzheimer's disease should be based on impairment of hippocampal type memory,^18^ as disclosed by memory tests that include controlled learning and cued recall, confirmed by AD biomarkers. 

The MBT aims to improve early detection of memory disorders and decline when memory is still within the normal range, while overcoming some limitations of other tests. These limitations include: a) memory units, b) associative binding, c) maximum retrieval, and d) early detection of memory disorders. 

In conclusion, early detection in the pre-dementia stage of Alzheimer's disease and other dementias is needed not only for clinical application but also as an instrument to assess clinical trials, evaluate the effectiveness of early interventions, and to select individuals for AD biomarker studies.

Since forgetfulness is often the earliest and most prominent feature in Alzheimer's disease, a test to detect memory impairment should be brief, easy to repeat and low cost to allow the detection of memory impairment at the pre-symptomatic stage.

The MBT is a useful tool in the detection of MCI, constituting a simple, easily administered test that places low burden on the patient. Statistical analyzes indicated that the operating characteristics of the MBT, as well as its sensitivity, specificity, positive predictive value and negative predictive value are well suited, surpassing other tests commonly used for detecting MCI.
